# Daily Magnesium Intake and Serum Magnesium Concentration among Japanese People

**DOI:** 10.2188/jea.JE2007381

**Published:** 2008-08-07

**Authors:** Yoriko Akizawa, Sadayuki Koizumi, Yoshinori Itokawa, Toshiyuki Ojima, Yosikazu Nakamura, Tarou Tamura, Yukinori Kusaka

**Affiliations:** 1 Chiyoda Public Health Center; 2 Fukui National College of Technology; 3 Jinai Women’s College; 4 Department of Social Health of Medicine, Hamamatsu Medical University; 5 Department of Public Health, Jichi Medical University; 6 Department of Internal Medicine, National Hospital Organization, Kinki-Chuo Chest Medical Center; 7 Department of Environmental Health School of Medicine, Fukui University

**Keywords:** Random Sampling, National Nutrition Survey, Daily Magnesium Intake, Serum Magnesium Concentration, Spectrum Analysis

## Abstract

**Background:**

The vitamins and minerals that are deficient in the daily diet of a normal adult remain unknown. To answer this question, we conducted a population survey focusing on the relationship between dietary magnesium intake and serum magnesium level.

**Methods:**

The subjects were 62 individuals from Fukui Prefecture who participated in the 1998 National Nutrition Survey. The survey investigated the physical status, nutritional status, and dietary data of the subjects. Holidays and special occasions were avoided, and a day when people are most likely to be on an ordinary diet was selected as the survey date.

**Results:**

The mean (±standard deviation) daily magnesium intake was 322 (±132), 323 (±163), and 322 (±147) mg/day for men, women, and the entire group, respectively. The mean (±standard deviation) serum magnesium concentration was 20.69 (±2.83), 20.69 (±2.88), and 20.69 (±2.83) ppm for men, women, and the entire group, respectively. The distribution of serum magnesium concentration was normal. Dietary magnesium intake showed a log-normal distribution, which was then transformed by logarithmic conversion for examining the regression coefficients. The slope of the regression line between the serum magnesium concentration (Y ppm) and daily magnesium intake (X mg) was determined using the formula *Y* = 4.93 (log_10_*X*) + 8.49. The coefficient of correlation (r) was 0.29. A regression line (*Y* = 14.65*X* + 19.31) was observed between the daily intake of magnesium (Y mg) and serum magnesium concentration (X ppm). The coefficient of correlation was 0.28.

**Conclusion:**

The daily magnesium intake correlated with serum magnesium concentration, and a linear regression model between them was proposed.

## INTRODUCTION

Presently, in Japan, health food products, including dietary supplements, are treated merely as conventional foods, and there are no laws to regulate their production and consumption. They are subjected to myriad regulations, such as those concerning food safety and food hygiene; the Drugs, Cosmetics and Medical Instruments Act; and rules related to the standardization of agriculture and forestry supplies and adequacy of quality control methods. No government agency controls these products in a systematic manner from their production to sale, managing the damages they may cause. When an adverse effect of such a product on health is discovered, the Safety Committee of the Cabinet makes decisions in a case-by-case manner. Thus, in the case of an accident, people are expected to assume the responsibility of the outcome.

Despite this legal inadequacy, the market for these so-called health foods is mushrooming due to the current interest in health promotion.^1^ According to the result of a survey, i.e., “The awareness and behavior of housewives concerning health foods,” conducted by the National Life Center in 2005, 1 out of 4 individuals consumed these health foods almost on a daily basis. The expenditure incurred on these foods increases with age: more than 10% of individuals in their 50s and 60s were shown to spend >10,000 yen, and some even spent >50,000 yen per month.^2^

It is suspected that a considerable number of them are unaware of the type or quantities of nutrients that they are consuming; under the influence of mass media advertising campaigns that extol the virtues of health foods containing magnesium, they blindly believe that they are suffering from a magnesium deficiency and enthusiastically consume such health foods with the hope of gaining the proclaimed health benefits.

A television station addressed the subject of the beneficial effects of consuming magnesium. The body fat-lowering effect of magnesium was illustrated in an experiment in which the effect of the presence or absence of magnesium on combustion of fat (substituted by an oil from an animal source) was observed. In the same program, a weight-loss method was introduced wherein one was shown that one could lose weight by drinking a juice containing *nigari* (bittern), which was advertised to be a simple means of consuming magnesium.

According to a Mainichi newspaper article dated July 16, 2004, since late 2003, 2 patients are reported to have been brought to the Tokyo Metropolitan Bokuto Hospital Emergency Center with cardiopulmonary arrest due to acute magnesium intoxication: one of them was a man in his 70s who consumed approximately 100 mL of nigari (containing magnesium) from a PET bottle when his family recommended the use of a small amount of it to treat his constipation and the other was a woman in her 40s who consumed approximately one-half of the contents of a PET bottle of nigari and was brought to the emergency room minutes before cardiac arrest. Fortunately, treatment by hemodialysis saved their lives.

Some nutritionists teaching at universities ignore the importance of understanding (1) the properties of each nutrient and (2) the quantity of that nutrient that a healthy adult should consume daily. They lecture on the need for using nutritional supplements without emphasizing on the importance of correcting poor lifestyle habits, such as overeating, nutritional imbalance, excessive consumption of alcoholic beverages, and irregular eating habits.^3^^,^^4^

The vitamins and minerals that are deficient in the daily diet of a normal adult remain unknown. As part of the answer to this question, we decided to focus on the relationship between dietary magnesium and serum magnesium level. Magnesium plays an important role in stabilizing adenosine triphosphate and other molecules.^5^ It is also one of the essential mineral elements.^6^ In human beings, magnesium contained in the normal diet is absorbed through the intestinal tract. The mechanism of intestinal absorption of magnesium has not been completely elucidated.^5^ In adults, approximately 50-60% of the magnesium is contained in the bones, which are known to be a site of magnesium storage. During periods of magnesium deficiency, the bones release it so that it can be used as required in other parts of the body. The parathyroid hormone is presumed to be involved in this process; however, the details of this process remain unknown.^5^

It has been suggested that if the deficiency in magnesium intake (however small in magnitude) persists for a long time, the risk of lifestyle-related diseases such as osteoporosis, heart disease, and diabetes mellitus increases; however, the mechanisms by which this occurs remain controversial.^5^ Some of the more recent epidemiologic studies indicate the relationship between the development of colon cancer and deficient magnesium intake.^7^ Studies on the dynamics of magnesium metabolism in vivo in the general population are scarce.^8^

Among the Japanese, who are known to have the world’s longest life expectancy, we observed the inhabitants of Fukui Prefecture, whose life expectancy is the longest among the Japanese^9^. We believe that it is important to observe the relationship between their dietary magnesium intake and serum magnesium level.

The present study was conducted on a group of subjects who were selected by stratified sampling from randomly selected areas to observe the following: (1) the distribution of magnesium intake stratified by sex and age, (2) serum magnesium concentration and its distribution, and (3) the relationship between magnesium intake and serum magnesium concentration. We also investigated the possibility of estimating magnesium intake from the serum magnesium concentration.

## METHODS

The subjects of the Comprehensive Survey of Living Conditions of the People on Health and Welfare 1998, were the households (approximately 280,000) or members of the households (approximately 780,000) located within 5,240 area units that had been randomly chosen from those selected by the Comprehensive Survey of Living Conditions of the People on Health and Welfare 1998. The subjects of the 1998 National Nutrition Survey were the households (approximately 5,000) or members of the households (approximately 15,000) located within 300 area units that had been randomly chosen from those selected by the 1998 National Survey on Basic Life Statistics (the subjects of the 1995 National Census of Japan). Among these households, 47 were located in Fukui Prefecture (Ohno-shi and Harue-machi); of these, 5 households were excluded because the individuals did not provide their consent and some of them were registered as foreign nationals, and the 156 members from the remaining 42 households served as the subjects of the 1998 Prefectural Nutrition Survey for Fukui Prefecture as well. The survey procedure composed of physical measurements (body height, body weight, blood pressure, hematological data, and oral health data collected by questioning), nutritional status, and dietary data. Blood investigations were not conducted on a minority of the National Nutrition Survey population, which comprised individuals <20 years old and infants; accordingly, these subjects were not included in this study.

Of the 156 persons living in the target area of the National Nutrition Survey, 62 provided us with verbal consent for the use of both their dietary data and the data on serum parameters; based on the consent of the individuals, the Fukui Prefectural Government also approved the use of the data. Therefore, we analyzed 62 participants (31 males and 31 females from 32 households); we were unable to perform physical examination of one of the study subjects. The Japanese government, which is responsible for conducting the National Nutrition Survey, contacted each local prefectural government to survey the residents. The local governments are permitted to use the data thus obtained for local policy making. The Fukui Prefectural Government approved of our study on the analysis of the relationship between dietary magnesium intake and serum magnesium levels. Thus, the Fukui Prefectural Government provided us with the data obtained from the residents who consented to participate in our study. The participants of the study group provided their verbal consent to the Fukui Prefectural Government (Public Health Center) for the 1998 National Nutrition Survey and to the authors of this study for using the data obtained by the local government in our survey. In order to obtain this consent, we also attended the survey conducted by the local government and explained to the participants the purpose of our research.

Prior to the study, its intent was thoroughly explained to all the members of the Public Health Center in order to provide them with a complete understanding of the survey procedure. At the time of the area-wise briefing session of the 1998 National Nutrition Survey in Fukui in October 1998, the subjects were explained the purpose of this study by the public health center staff and the authors of this study. In addition, informed consent was obtained from these subjects, permitting the contents of the National Nutrition Survey questionnaire (including physical measurements, nutrient intake, and dietary habits) to be made available to us by the public health center staff.

Holidays and special occasions were avoided, and a day when people were most likely to be on an ordinary diet was selected as the survey date (November 5, 1998, for Ohono-shi, and November 9, 1998, for Harue-machi). Prior to the survey, the purpose of the study was adequately explained to the participants. The survey sheet on food intake was distributed to each household; the method for entering the data was thoroughly explained; and each food item was weighed using a scale, and the weight was entered into the survey sheet. When the amount was too small to be weighed, a rough estimate of the amount was entered. One person in each household weighed and recorded the cooked dishes and the ingredients of each dish, the amount of food consumed and wasted, and the approximate proportions consumed by each family member. The type and amount of foods consumed outside the home were also included when recording an individual’s food consumption.

In addition, a dietitian, who was also a member of the survey team, personally visited each household, verified the entered data, made corrections, or instructed the subject on how to record the data correctly. The intake of additional dietary supplements, such as those containing vitamins or minerals, was not examined.

A card was prepared for each subject based on the intake listed in the food intake survey sheet. It depended on the proportion of food consumed by an individual within each household, which was recorded for each family member by the home manager. We used the fifth revised edition of the Standard Tables of Food Composition in Japan,^10^ which is based on the values of magnesium intake measured by an atomic absorption spectrometer (AAS).^11^

On the day on which the physical measurements were conducted (November 4, 1998, in Ohno-shi, and November 11, 1998, in Harue-machi), 5 mL of blood was collected from each subject using a vacuum tube; the serum was then separated (by centrifugation at 1 atm pressure and 15°C) and immediately stored at a temperature below -40°C.

Following dissolution, the serum was diluted 10 times with 0.001 mol/dm^3^ nitric acid to which 5 μg/mL of yttrium (Wako Pure Chemical Industries Ltd., Osaka, Japan) had been added. A quantitative analysis was conducted using an OPTIMA 3000 inductively coupled plasma (ICP) atomic spectrometer (Perkin Elmer Inc., Waltham, Massachusetts, USA; magnesium detection limit, 0.1 ppb). The magnesium concentration of the same sample was also examined using an AAS manufactured by the same company (4100ZL; magnesium detection limit, 0.008 ppb) to confirm the accuracy of the result obtained by ICP spectrometry. The serum magnesium values obtained by ICP spectrometry were used for computation since these values in the order of 10 ppb were equal to the values obtained by atomic absorption spectrometry.

Excel^®^ 2003 (Microsoft) was used for statistical analysis and testing. The normality of the histogram was examined using a chi-square conformity test, in which the chi-square values indicating the observed frequencies and their deviations from the expected values are used. If a logarithmic distribution was noted instead of a normal distribution, it was appropriately transformed. The Student’s *t* test was used for the mean statistics, and the correlation was examined using Pearson’s correlation coefficient test. The significance level was set at 5%.

## RESULTS

The profiles of the study subjects according to their sex, daily magnesium intake, magnesium intake per kilogram body weight (BW), and serum magnesium concentration are shown in Table 1.

**Table 1.** Profiles of the study subjects according to sex, magnesium intake per day, magnesium intake per kilogram body weight, and serum magnesium concentration

	All (n = 62)			Males (n = 31)			Females (n = 31)*		
		
	n (%)	Mean ± SD	Range	n (%)	Mean ± SD	Range	n (%)	Mean ± SD	Range
Age (year)		51.0 ± 17.0	20-90		48.0 ± 18.0	20-90		53.0 ± 16.0	26-89
Weight (kg)*		58.2 ± 11.3	35.0-84.0		63.3 ± 9.5	41.5-84.0		52.9 ± 10.6	35.0-72.5
Body mass index (kg/m^2^)*		22.4 ± 3.1	15.9-30.8		22.7 ± 2.4	17.4-29.1		22.2 ± 3.8	16.0-31.0
Consumption of antihypertensive medicine*	3 (5)			0 (0)			3 (10)		
Current smoking status*	20 (33)			16 (52)			4 (13)		
Daily alcohol consumption*	11 (18)			10 (32)			1 (3)		
Habitual exercise*	11 (18)			6 (19)			5 (17)		

Intake of magnesium (mg/day)		322 ± 147	143-950		322 ± 132	143-861		323 ± 163	152-950
Intake of magnesium per kilogram body weight (mg/kgBW/day)*		5.75 ± 2.75	2.20-14.50		5.22 ± 2.44	2.20-13.89		6.30 ± 2.99	2.68-14.50

Serum magnesium concentration (ppm)		20.69 ± 2.83	13.08-28.90		20.69 ± 2.83	15.18-28.90		20.69 ± 2.88	13.08-26.65

* : Of the 31 females, 30 were examined for physical status.

SD: standard deviation

The mean daily magnesium intake was 322 ± 132 mg/day for men (n = 31), 323 ± 163 mg/day for women (n = 31), and 322 ± 147 mg/day for the entire group (n = 62). The mean daily magnesium intake per kilogram BW was 5.22 ± 2.44 mg/kg/day for men (n = 31), 6.30 ± 2.99 mg/kg/day for women (n = 31), and 5.75 ± 2.75 mg/kg/day for the entire group (n = 62). The mean serum magnesium concentration was 20.69 ± 2.83 ppm for men (n = 31), 20.69 ± 2.88 ppm for women (n = 31), and 20.69 ± 2.83 ppm for the entire group (n = 62). No significant difference was observed in the mean serum magnesium concentration between men and women.

**Figure 1.**
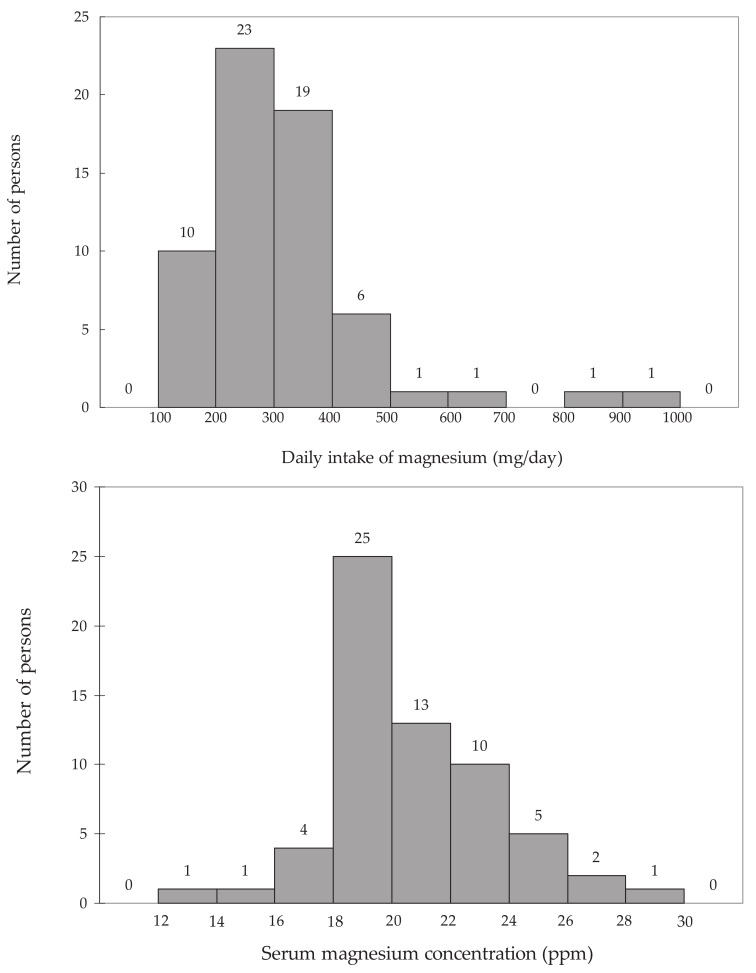
Histogram representation of the distribution of daily intake of magnesium and serum magnesium concentration

It was observed that sex had no effect on the daily magnesium intake or on the daily magnesium intake per kilogram BW, when the difference was examined using the Student’s *t* test. Magnesium intake was expressed in the form of a histogram (Figure 1). Normality was examined using a chi-square goodness-of-fit test in which the chi-square values represent the frequencies of the observations and their deviations from the expected values. When examined for normality, the chi-square value was 9.95 (degree of freedom (df) = 8), and a normal distribution was not noted at a significance level of 5%. Next, a logarithmic conversion was performed to examine normality. When a logarithmic conversion was used to test for normality, the chi-square value was 2.32 (df = 8), and a normal distribution was noted at a significance level of 5%. The distribution of serum magnesium concentrations is shown in Figure 1. Normality was examined by a chi-square goodness-of-fit test where the bias between the observed frequency and the expected value was examined by a chi-square value. The chi-square value was found to be 4.53 (df = 8), and a normal distribution was noted at a significance level of 5%.

Because of the limited number of specimens available and the lack of evidence for a sex-related difference in the serum magnesium concentrations and dietary magnesium intake, male and female samples were pooled to observe the relationship between dietary magnesium intake and its serum concentration. The slope of the regression line between the serum magnesium concentration (Y ppm) and daily dietary magnesium intake (X mg) was determined using the formula *Y* = 4.93 (log_10_*X*) + 8.49. The coefficient of correlation (r) between the serum magnesium concentration and daily magnesium intake was determined to be 0.29 at a significance level of 5%. Further, the slope of the regression line between the daily dietary magnesium intake (Y mg) and serum magnesium concentration (X ppm) was determined using the formula *Y* = 14.65*X* + 19.31. The coefficient of correlation (r) between the daily magnesium intake and serum magnesium concentration was observed to be 0.28 at a significance level of 5%. Thus, serum magnesium concentration was observed to correlate with the daily dietary magnesium intake.

## DISCUSSION

The recommended magnesium intake according to the international standards is 4.5 mg/kg BW/day.^12^ Although in this study, we determined the daily magnesium intake based on only a 1-day meal record, this was calculated to be 5.75 ± 2.75 mg/kg BW/day. Compared with the international standards, these subjects obtain sufficient magnesium from their daily meals. Peacock et al.^13^ computed the magnesium intake assessed from a food frequency questionnaire and stratified it by the serum magnesium concentration. They found that the mean daily magnesium intake among women and men was 241-252 and 270-279 mg/day, respectively. Our data indicate that the intake among women and men is 323 ± 163 mg/day (range, 152-950) and 322 ± 132 mg/day (range, 143-861), respectively. It was concluded that the mean daily dietary magnesium intake is fairly high among healthy subjects. Although there are no differences in the measured values resulting from the different investigation methods used, a difference resulting from the differences in dietary habits is believed to exist.

The National Nutrition Survey was conducted in 1998, and the consumption of nutritional supplements, such as those containing vitamins or minerals, was not examined. Then, the intake of nutritional supplements was examined only by a questionnaire on dietary habits included in the 2001 National Nutrition Survey. The results of this questionnaire survey showed that 83% of the men and 62% of the women did not consume any nutritional supplements. In addition, among the individuals who consumed supplements, only 8% of the men and 10% of the women were shown to consume supplements containing magnesium. Accordingly, for the purpose of the current study, it was supposed that the data from the year 1998, when magnesium supplements were hardly consumed, would be accurate enough to examine magnesium intake from 1 meal alone.

In the fifth Standard Table of Food Composition in Japan (2001), the magnesium contents in different types of foods consumed by the Japanese were described.^10^ The 2001 National Nutrition Survey^14^ examined the daily dietary magnesium intake in the healthy Japanese population by adopting the same method that has been used in the current study, and determined the mean daily dietary magnesium intake to be 272 ± 107 mg/day among all the subjects (n = 9,825; all the subjects were aged >20 years), 287 ± 111 mg/day among men (n = 4,507), and 258 ± 102 mg/day among women (n = 5,318). The data in the current study is larger than that of the 2001 National Nutrition Survey. The difference in the calendar years (1998 and 2001) and regions may explain these discrepancies. In this study, no sex-related difference was observed with regard to the quantity of magnesium intake. In contrast to the findings of the 2001 National Nutrition Survey, the mean daily dietary magnesium intake was slightly higher among women as compared to that in men, unlike that observed in this study. Magnesium is abundant in fruits and vegetables; however, in refined and processed foods or the so-called instant foods, it is present in negligible amounts.

Taking together the results of a magnesium balance test for Japanese^15^ and Americans,^16^ the Ministry of Health, Labour, and Welfare of the Japanese government published the “Dietary Reference Intakes for Japanese” in 2005.^17^ The estimated average requirement (EAR) of magnesium per day among Japanese men was 290 mg/day in the age group 18-29 years, 310 mg/day in the age group 30-49 years, 290 mg/day in the age group 50-69 years, and 260 mg/day for age ≥70 years. The EAR of magnesium per day among Japanese women was 230 mg/day in the age group 18-29 years, 240 mg/day in the age group 30-69 years, and 220 mg/day for age ≥70 years. The recommended dietary allowances (RDAs) based on the food consumption determined by the Japanese Ministry of Health, Labour and Welfare^17^ are 340, 370, 370, 350, and 310 mg/day for men between 18 and 29 years of age, between 30 and 49 years of age, between 50 and 69 years of age, and ≥70 years of age, respectively; they are 270, 280, 290, and 270 mg/day for women between 18 and 29 years of age, between 30 and 49 years of age, between 50 and 69 years of age, and ≥70 years of age, respectively. Among the subjects of the current study, a total of 20 (32%) subjects, i.e., 12 men (39%) and 8 women (26%) did not fulfill the RDA.

In this study, determination of the quantity of magnesium intake depended on the daily meal records. This is because we conformed to the protocol of the National Nutrition Survey (1998). However, there are certain problems associated with this protocol, which considers the magnesium intake only for a day and not according to the subject’s custom. Accordingly, there are limitations to the result thus obtained. A few individuals may be unable to achieve a dietary intake as per the RDA. If we investigate the meal records over a longer period of time, the correlation between serum magnesium concentration and daily magnesium intake would become more evident; therefore, such a study is desirable.

There are several methods of measuring the serum magnesium level: the EDTA method, flame spectrophotometry, the Titan yellow method, ICP spectrometry, and enzyme analysis. Currently, atomic absorption spectrophotometry is used most frequently because of its reproducibility, simplicity, and sensitivity.^18^ In the current study, ICP and atomic emission spectrophotometry were used concurrently. ICP spectrometry is easier to perform and is considered more desirable in the future for the analysis of multiple samples. The mean serum magnesium concentrations have been reported previously by Khan et al. (0.73 ± 0.03 mmol/L (17.52 ± 0.72 ppm))^19^ and by Abraham et al. (1.7 ± 0.04 mg/100 mL (17.0 ± 0.40 ppm)), ^20^ based on atomic absorption spectrophotometric measurements in healthy individuals; although we followed the same method in the present study, the serum magnesium concentration values obtained were higher. It may be correct to state that the serum magnesium concentration in the normal Japanese population is higher than that found in other populations.

The questionnaire on dietary intake that was used in the 1998 National Nutrition Survey^21^ did not include questions on the use of nutritional supplements by people. Based on the statistical distribution of the serum magnesium level in this study, it is unlikely that there were individuals who consumed large quantities of magnesium supplements that would have caused notable fluctuations in the results. We applied the average daily dietary magnesium intake among adults from the 2001 National Nutrition Survey to the formula *Y* = 4.93 (log_10_*X*) + 8.49 and estimated the serum magnesium concentration. It is predicted to be 20.49, 20.61, and 20.38 ppm for the overall subjects, men, and women, respectively. The standard serum magnesium concentration among the healthy Japanese population is listed as 1.8-2.3 mg/dL (0.74-0.95 mmol/L) (18-23 ppm),^22^ while according to the Current Medical Diagnosis and Treatment, it is listed as 1.5-2.5 mEq/L (18-30 ppm).^23^ According to these sources, the normal values vary over a wide range. Among the subjects of the current study, the serum magnesium concentration in 6 individuals (9.7%)-3 men and 3 women-did not reach the standard value of 18 ppm. A study conducted by Tsunematsu showed that sex and age had no effects on serum magnesium concentration among healthy persons, the mean ± standard deviation of total serum magnesium concentration being 1.76 ± 0.12 mEq/L (21.0 ± 1.44 ppm).^8^ Because of the limited number of samples in this study, it was not possible to detect age-related fluctuations. Moreover, sex-related differences were also not detected as reported by Tsunematsu. In this study, the mean ± standard deviation of the total serum magnesium concentration was 20.69 ± 2.83 ppm, which was consistent with that reported by Tsunematsu.^8^

According to Peacock et al., the coefficient of correlation between serum magnesium concentration and daily magnesium intake was 0.053, thus indicating no correlation between the 2 factors.^13^ However, the coefficient of correlation computed in the current study was 0.29. When tested for significance, the correlation was shown to be significant at a significance level of 5%. It may be noteworthy that the difference between these 2 values is due to the difference between the methods of measurement of the daily magnesium intake. We constructed a scattergram by plotting the serum magnesium concentrations (ppm) on the Y-axis and dietary magnesium intake (mg/day) converted to the logarithmic scale on the X-axis, which yielded a linear regression equation, i.e., *Y* = 4.93 (log_10_*X*) + 8.49. Further, a linear regression between the daily magnesium intake (Y mg) and serum magnesium concentration (X ppm) was calculated to be given by the equation *Y* = 14.65*X* + 19.31. Thus, it was suggested that magnesium intake can be estimated based on the serum magnesium concentration. By employing a model that enables the estimation of magnesium intake from the serum magnesium level, the required optimum serum magnesium concentration for a healthy individual can be determined. Thus, it may be possible to determine the individual dietary magnesium requirements during nutritional counseling in order to prevent lifestyle-related diseases, improve one’s health, forestall aging, and avert an excess or deficiency of magnesium.

In our study, we included individuals belonging to the same household, considering every individual as an independent sample. However, the meal intake for individuals belonging to the same household may be similar. This implies that sampling either the entire household or 1 family member leads to different distributions of dietary magnesium intake or serum magnesium concentrations. To study this possibility, in a separate study, we selected 1 individual from each household and compared their mean serum magnesium concentration and mean daily dietary magnesium intake with the results of the entire study population. We randomly selected 1 individual from each household and formed a new subgroup of 32 individuals (16 males and 16 females). The mean serum magnesium concentration of the entire population (n = 62) was equal to that of the new subgroup (n = 32), i.e., 21.07 ± 2.66 ppm (21.16 ± 2.97 ppm for males and 20.98 ± 2.41 ppm for females). (The officially approved difference in average is at dangerous rate of 5%.) The mean daily magnesium intake among the entire study group (n = 62) was comparable with that of the new subgroup (n = 32), i.e., 334 ± 157 mg (311 ± 73 mg for males and 356 ± 211 mg for females). Moreover, the values for the entire groups and females were identical (The officially approved difference in average is at a dangerous rate of 5%.); however, in the case of males, this was not observed. The coefficient of correlation between the entire study group and the entire subgroup was 0.06. Therefore, considering all the subjects as independent specimens may have led to this discrepancy. Sampling of a greater number of individuals from a greater number of households is thus required.

More studies employing a larger number of test subjects must be conducted before the formula suggested in this study can be utilized on a larger scale. For example, if the data regarding an individual’s serum magnesium level is added to the biochemical database of the National Health and Nutrition Survey conducted by the Ministry of Health, Labour and Welfare, a greater number of specimens will become available and a larger dataset will be available for analysis. As a result, a clear-cut relationship between the serum magnesium level and magnesium intake can be determined, and a formula to adequately express it can be deduced. By establishing the relationship between magnesium intake and serum magnesium level, the former can be readily deduced from the latter, thereby circumventing cumbersome and expensive procedures. By detecting insufficient or excessive dietary magnesium intake during nutritional counseling, one can prevent cancers; cardiovascular diseases such as heart disease and hypertension; metabolic diseases such as hyperglycemia, hyperlipidemia, and hyperuricemia; and lifestyle-related diseases such as osteoporosis. The results of this study will contribute to further studies on the relationship between dietary magnesium intake and serum magnesium level in order to elucidate the effect of this mineral element on lifestyle-related diseases such as cardiovascular diseases.

## References

[1] Osaka N, Fujio A, Tsutsui M, Miyata H, Hirose Y, Ishikawa M, et al. They step on healthy boom and grow rapidly, supplement blooms in profusion. Weekly Toyo Keizai. 2005 January 4; p. 26-44.

[2] National Consumer Affairs Center of Japan. The awareness and behavior of housewives concerning health foods, 2005. [cited 2006 Nov 3]. Available from: http://www.kokusen.go.jp/pdf/n-20050304_2.pdf.

[3] Nakamura T Nutritional unbalance intake and dietary supplement. J Clin Exp Med (Igaku No Ayumi) 2004; 208(12):975-7.

[4] Nakamura T. A supplement to understand well, in order to take a vitamin well. In: Hashizume N, editor. Vitamin supplement guidebook for experts. Tokyo: Ishiyaku Publishers Inc. Press; 2003. p. 141-50.

[5] Fleet JC, Cashman KD. Magnesium. In: Bowman BA, Russell RM, editors. Present knowledge in nutrition. 8th ed. Washington DC: ILSI Press; 2001. p. 292-301.

[6] Suzuki K. Nourishment of a mineral. In: Hayashi J, editor. N Books, Basic science of nutrition. Tokyo: Kenpakusha Co. Press; 2004. p. 119-29.

[7] Folsom AR, Hong CP. Magnesium intake and reduced risk of colon cancer in a prospective study of women. Am J Epidemiol 2006;163:232-5. 10.1093/aje/kwj037 16319289

[8] Tsunematsu K, Tanuma S, Sakuma Y Lymphocyte Mg values in Japanese determined by the micro-analysing method. Jpn J Magnes Res 1987;6:33-43.

[9] Japan Health Sciences Research Grants by the Ministry of Health and Welfare. Fukui Centenarian Study. Kusaka Y, editor. Fukui: Printing Craster Coop. Press; 1998.

[10] Japan Department of Science and Technology Agency, Japan. Standard tables of food composition in Japan. 5th revised ed. Tokyo: Japan National Printing Bureau; 2000.

[11] Japan Food Research Laboratories. In: An analysis business person wrote a commentary of standard tables of food composition in Japan. 5th revised ed. Analysis Manual. Chuohoki Co. Ltd. Press; 2001. p. 110-1.

[12] Dreosti IE. Magnesium status and health. In: Rosenberg IH, editor. Nutrition Reviews vol. 53, No. 9 (Part II) The Kellogg Nutrition Symposium Micronutrients: their role in a modern lifestyle. Tokyo: Kenpakusha Co. Press; 1996. p. 33-9.

[13] Peacock JM, Folsom AR, Arnett DK, Eckfeldt JH, Szklo M. Relationship of serum and dietary magnesium to incident hypertension: the Atherosclerosis Risk in Communities (ARIC) Study. Ann Epidemiol 1999;9:159-65. 10.1016/S1047-2797(98)00040-4 10192647

[14] Ministry of Health, Labour and Welfare, Japan. The National Nutrition Survey in Japan, 2001. Japan Government Printing Office; 2003.

[15] Nishimuta M, Kodama N, Yoshioka YH, Morikuni E. Magnesium intake and balance in the Japanese population. In: Rayssiguier Y, Mazur A, Durlach J, editors. Advances in magnesium research: nutrition and health. UK: John Libbey and Company Ltd.; 2001. p. 197-200.

[16] Jones JE, Manalo R, Flink EB. Magnesium requirements in adults. Am J Clin Nutr 1967;20:632-5. 5338728 10.1093/ajcn/20.6.632

[17] Ministry of Health, Labour and Welfare, Japan. Dietary Reference Intake for Japanese, 2005. Japan Government Printing Office; 2004.

[18] Tanuma S. The measurement method and how to read of test value of magnesium. In: Itokawa Y, editor. Magnesium, relation with life-style related diseases. Tokyo: Koseikan Co. Press; 1995. p. 49-69.

[19] Khan LA, Alam AM, Ali L, Goswami A, Hassan Z, Sattar S, Serum and urinary magnesium in young diabetic subjects in Bangladesh. Am J Clin Nutr 1999;69:70-3. 9925125 10.1093/ajcn/69.1.70

[20] Abraham GE, Lubran MM. Serum and red cell magnesium levels in patients with premenstrual tension. Am J Clin Nutr 1981;34:2364-6. 7197877 10.1093/ajcn/34.11.2364

[21] Ministry of Health, Labour and Welfare, Japan. The National Nutrition Survey in Japan, 1998. Japan Government Printing Office; 2000.

[22] Terasawa A. Magnesium. In: Kanai M, editor. Kanai’s manual of clinical laboratory medicine. Tokyo: Kanehara Co. Press; 2005. p. 574-7.

[23] Fukagawa M, Kurokawa K, Papadakis MA. Electrolyte bodily fluids and abnormality. In: Tierney LM Jr, McPhee SJ, Papadakis MA, editors. Current medical diagnosis & treatment 2004. 43rd ed. Nittkei BP, Tokyo: Dainihoninsatu Press; 2004. p. 878.

